# Importance of the ammonia assimilation by *Penicillium purpurogenum* in amino derivative *Monascus* pigment, PP-V, production

**DOI:** 10.1186/2191-0855-3-19

**Published:** 2013-03-28

**Authors:** Teppei Arai, Kasumi Koganei, Sara Umemura, Ryo Kojima, Jun Kato, Takafumi Kasumi, Jun Ogihara

**Affiliations:** 1 Department of Chemistry and Life science, College of Bioresource Sciences, Graduate School of Bioresource Sciences, Nihon University, 1866 Kameino, Fujisawa, Kanagawa, 252-0880, Japan

**Keywords:** *Monascus* pigment, Amino derivative, *Penicillium purpurogenum*, Ammonia assimilation, Glutamine synthetase, Glutamate dehydrogenase

## Abstract

A fungal strain, *Penicillium purpurogenum* IAM 15392, produced the azaphilone *Monascus* pigment homolog when cultured in a medium composed of soluble starch, ammonium nitrate, yeast extract, and citrate buffer, pH 5.0. One of the typical features of violet pigment PP-V [(10*Z*)-12- carboxyl-monascorubramine] is that pyranoid oxygen is replaced with nitrogen. In this study, we found that glutamine synthetase (*glnB*) and glutamate dehydrogenase (*gdh1*) genes were expressed in the culture conditions conducive to PP-V production. Gln and Glu both support PP-V biosynthesis, but PP-V biosynthesis was much more efficient with Gln. We determined that synthesis of Gln by glutamine synthetase from ammonium is important for PP-V production.

## Introduction

Since ancient times, *Monascus* spp. have been used as starter cultures in the brewing of red rice wine, and *Monascus* pigments have been used as a natural food colorant in East Asia (Juzlova et al. 1996 ; Dufosse 2006). *Monascus* pigments comprise six major compounds, including the yellows of monascin (Birch et al. 1962 ; Chen et al. 1971 ; Fielding et al. 1961) and ankaflavin (Manchand et al. 1973), oranges of monascorubrin (Hadfield et al. 1967 : Kurono et al. 1963) and rubropunctatin (Haws and Holker 1961), and reds of monascorubramine (Hiroi et al. 1975) and rubropunctamine (Fowell et al. 1956). Pigments produced by *Monascus* spp. were recently demonstrated to have physiological activities as antibacterial, anticancer, and antioxidant agents (Kim et al. 2006 ; Akihisa et al. 2005 ; Akihisa et al. 2005). However, except for a few strains that have been developed for commercial use, *Monascus* spp. have also been found to produce citrinin, which is a mycotoxin (Blane et al. 1995 ; Wang et al. 2005). For this reason, *Monascus* pigments are not approved as food ingredients in the European Union or the United States.

*Penicillium purpurogenum* IAM 15392 has been found to produce *Monascus* pigment homologs in culture with a specific medium (Ogihara et al. 2000 ; Ogihara et al. 2001 ; Ogihara and Oishi 2002). This strain does not produce citrinin, making *P. purpurogenum* IAM15392 a potentially valuable commercial source of natural food colorant.

Many of the filamentous fungus *Penicillium* spp. are known to produce red pigments that contain quinone and phenolic compounds (Takeda et al. 1973 ; Gatenbeck 1959 ; Bu’Lock and Smith 1968 ; Cason et al. 1962 ; Simonart and Verachtert 1966 ; Fuska et al. 1988 ; Peterson and Grove 1983 ; Singh et al. 1985 ; Kobayashi et al. 1987). *P. purpurogenum* IAM 15392 has been found to produce *Monascus* pigment homologs PP-V [(10*Z*)-12-carboxyl-monascorubramine] and PP-R [(10*Z*)-7-(2-hydroxyethyl)-monascorubramine] in culture with a medium composed of ammonium nitrate, soluble starch, yeast extract, and citrate buffer at pH 5.0. In an ammonium nitrate-free medium, PP-O [(10*Z*)-12-carboxyl-monascorubrin] and PP-Y [(10*Z*)-monascorubrin] pigment were produced instead of PP-V and PP-R, respectively (Ogihara et al. 2000 ; Ogihara et al. 2001 ; Ogihara and Oishi 2002). PP-V is a nitrogen-containing polyketide compound that is important as a secondary metabolite for microorganisms. The nitrogen atom in the nitro compound often exhibits various biological activities with medical and agrochemical applications. As a nitrogen source for PP-V production, culture of *P. purpurogenum* IAM15392 with ammonium nitrate results in an efficient and stable yield of the pigment. Based on the structural characteristics of 7-NH in PP-V, the role of ammonia nitrogen in pigment production was suggested.

*Monascus* spp. are reported to produce various derivatives of *Monascus* pigments with the supplementation of specific amino acids in culture broth. Glutamic acid, aspartic acid and alanine derivatives of *Monascus* pigment have been identified and characterized (Lin et al. 1992 ; Blanc et al. 1994 ; Hajjaj et al. 1997 ; Sato et al. 1997). Further, Jung et al. obtained various pigment derivatives using 20 amino acids as side chain precursors (Jung et al. 2003). The features of pigment production by *P. purpurogenum* IAM15392 include the production of PP-V, an amino derivative of the PP-O *Monascus* pigment homolog, when cultured in a medium supplemented with ammonium nitrate (Ogihara et al. 2000). However, little is known about the profile and characteristics of *Monascus* pigment derivatives produced in growth medium supplemented with inorganic nitrogen (Chen and Johns 1993).

The addition of ammonium ion with ammonium nitrate is important in PP-V production in culture broth, and NO_3_^-^ is used for PP-V production after reduction to NH_4_^+^ through an in situ bioprocess (Arai et al. 2012). We are therefore interested in the effect of the type of nitrogen source on incorporation into PP-V. Nitrogen, one of the most important biogenic elements, is incorporated into the cell in an inorganic (nitrate, nitrite, ammonia) or an organic (amino acids, urea, other nitrogen compounds) form. Ammonia is transported into the cell or forms a metabolite of nitrate or nitrite and is converted to amino acids by ammonia assimilation involving two principal enzymes: glutamate dehydrogenase (Gdh; EC 1.4.1.2 and EC 1.4.1.4) and glutamine synthetase (GS; EC 6.3.1.2). Fungal ammonium assimilation is reported to occur via incorporation into Glu and Gln by glutamate dehydrogenase A (GdhA) and GS (Hammond and Wood 1985 ; Casper et al. 1985 ; Dunn-Coleman et al. 1981 ; Limon-Lason et al. 1977).

In this study, we investigated the effect of nitrogen source on its incorporation into PP-V production. Expression of *GS* and *Gdh* genes, and inhibition of pigment production using a specific inhibitor of GS, L-methionine-DL-sulfoximine (MSX), were analyzed. We discuss how GS and Gdh contribute to ammonium assimilation and PP-V production.

## Materials and methods

### Fungal material

*P. purpurogenum* IAM 15392 was used in this study. A culture of *P. purpurogenum* IAM 15392 was deposited in the IAM Culture Collection, Institute of Molecular and Cellular Biosciences, The University of Tokyo and as JCM 23216 in the Japan Collection of Microorganisms, RIKEN Bioresource Center, Japan.

### Pigment production medium

One loopful of spores and mycelia of strain IAM15392 from a stock culture grown on YMA plates (10 g of glucose, 5 g of peptone, 3 g of yeast extract, 3 g of malt extract, and 20 g of agar per liter) was inoculated into a 500-ml Erlenmeyer flask containing 100 ml of a PP-V production medium (20 g of soluble starch, 2 g of yeast extract and 3 g of ammonium nitrate per liter of 50 mM citric acid/Na_3_ citrate buffer, pH 5.0) and PP-O production medium (20 g of soluble starch and 2 g of yeast extract per liter of 50 mM citric acid/Na_3_ citrate buffer, pH 5.0) and cultivated at 30°C with shaking at 200 rpm for 72 hr.

### 454 Sequencing and bioinformatic analysis

Genomic DNA was extracted from mycelium grown in stationary liquid culture and used in sequencing. Shotgun sequencing was performed at the Hokkaido System Science with the GS FLX Titanium system (Roche Diagnostics, Mannheim, Germany). The 454 sequencing reads were assembled into contigs with the GS De Novo Assembler software (Roche Diagnostics). The contigs were converted to Blast database format for local blast searches using stand-alone Blast software (ver. 2.2.22) downloaded from the NCBI website. Gene predictions were manually checked by comparing the sequences with homologous gene/proteins in the GenBank database. Functional domains in the translated protein sequences were predicted using Conserved Domain Search (NCBI).

### Characterization of *GS* and *Gdh* genes

To characterize *GS* and *Gdh* genes present in *P. purpurogenum* IAM15392, genomic sequences obtained by sequencing were used to search for homologous *GS* and *Gdh* gene sequences through a blastX search of NCBI. Primers for the amplification of *GS* and *Gdh* genes were designed based on the obtained consensus sequences (Table 1).

**Table 1:** Primers used for reverse transcription (RT)-PCR and real-time (qRT)-PCR

**Name**	**Primer sequence**
Reverse transcription PCR	
glnA-for	5^′^-GGCTTCTGCTCGGTGATCTT-3^′^
glnA-rev	5^′^-GACTGGGCCAGCTTATCCAC-3^′^
glnB-for	5^′^-GTCCTTGCGAGGGTATCGAG-3^′^
glnB-rev	5^′^-CGACCAGTCAGACGCTCATC-3^′^
gdh1-for	5^′^-TCCGACGATCTCAATGTTGG-3^′^
gdh1-rev	5^′^-CGCCGTCTACATCGATACCA-3^′^
gdh2-for	5^′^-AAAGTGAATATGGGTGGCGG-3^′^
gdh2-rev	5^′^-TCCAGTTGCTTCAGGTCGAA-3^′^
Real-time PCR	
glnB-RT-for	5^′^-GAGGGTATCGAGATGGGTGA-3^′^
glnB-RT-rev	5^′^-GGTGGAAGGAGATCTGGACA-3^′^
gdh1-RT-for	5^′^-GTGTTACCTCGCTAGACGGC-3^′^
gdh1-RT-rev	5^′^-TCTACTCGATAGCTTCGCCC- 3^′^

### RNA extraction and reverse transcription

*P. purpurogenum* IAM15392 cells cultured for 48 h in basal medium and basal medium containing NaNO_3_ were subjected to RNA extraction with FAvorPrepTM Plant Total RNA Mini kit (Favorgen Biotech, Ping-Tung, Taiwan). Total RNA (1.5 μg) was reverse transcribed with PrimeScript® Reverse Transcriptase (Takara Bio, Shiga, Japan) using dT 15 primer.

### Expression analysis of *GS* and *Gdh* genes

Expression analysis of *GS* and *Gdh* genes was examined by reverse transcription (RT)-PCR and real-time (qRT)-PCR. RT-PCR was performed using first-strand cDNA as the template using the primers designed for amplification of *GS* and *Gdh* shown in Table 1. The amplification was carried out with an initial denaturation at 95°C for 5 min, followed by 30 cycles of 95°C for 30 s, 55°C for 30 s, and 72°C for 30 s.

qRT-PCR was performed using LightCycler Fast Start DNA Master SYBR Green I with a LightCycler system (LightCycler 330, Roche Diagnostics). *P. purpurogenum* IAM15392 GAPDH was used as the reference gene for RT-PCR.

### Effect Gln and Glu on PP-V production

*P. purpurogenum* IAM15392 in medium for PP-O production supplemented with 1 to 15 mM L-Gln and L-Glu was cultured at 30°C with shaking at 200 rpm for 120 hr. The culture broth was centrifuged (3,000 rpm, 4°C, 10 min) and pigments in the resulting supernatant were extracted with EtOAc and subjected to silica gel TLC analysis.

### Purification of pigments containing Gln

For the purification of pigments, *P. purpurogenum* IAM15392 was cultured in PP-O production medium containing 1 to 15 mM L-Gln at 30°C with shaking at 200 rpm for 120 hr. After 120 hr, cultures were filtered through ADOVANTEC filter paper No. 2 (Toyo Roshi, Tokyo, Japan), and the pigment in the filtrate was extracted with EtOAc. The obtained extract was further purified by silica gel and Sephadex LH-20 column chromatography, as described previously (Ogihara et al. 2000).

### Pigment analysis

Pigments were detected by thin-layer chromatography using silica gel 60 F_254_ plates (Merck, Darmstadt, Germany) and developed in *n*-BuOH:AcOH:H_2_O (12:3:5) solvent. ^1^H and ^13^C NMR spectra were recorded using an ECA-500 spectrometer (JEOL, Tokyo, Japan).

### Effect of GS inhibition on pigment production

Concentration-dependent effects of GS-specific inhibitor L-methionine-DL-sulfoximine (MSX) were evaluated. *P. purpurogenum* IAM15392 was cultured in PP-V production medium containing 0, 1, and 2 mM MSX at 30°C with shaking at 200 rpm for 72 hr. The cultures were centrifuged (3,000 rpm, 4°C, 10 min) and pigments in the resulting supernatant were extracted with EtOAc and subjected to silica gel TLC analysis.

## Results

### Exploration of *GS* and *Gdh* genes

Two *GS* genes (*glnA*:AB746940 and *glnB*:AB746941) and two *Gdh* genes (*gdh1*:AB746942 and *gdh2*:AB746943) were identified from *P. purpurogenum* IAM15392 draft sequence data. These sequences showed high homology to the respective *GS* and *Gdh* genes of filamentous fungi (Table 2). The sequences has been submitted to GenBank database.

**Table 2:** **GS and Gdh gene in *Penicillium purpurogenum *IAM15392**

**Gene**	**Protein homolog (accession number)**	**Source of protein homolog**	**Identities**
*gln*A	glutamine synthetase, putative (XP 002480074.1)	*Talaromyces stipitatus* ATCC 10500	87%
	glutamine synthetase, putative (XP 002143731.1)	*Penicillium marneffei* ATCC 18224	83%
	glutamine synthetase (XP 747724.1)	*Aspergillus fumigatus* Af293	82%
*gln*B	glutamine synthetase (XP 002147563.1)	*Penicillium marneffei* ATCC 18224	87%
	glutamine synthetase (XP 002481714.1)	*Talaromyces stipitatus* ATCC 10500	86%
	glutamine synthetase (XP 001266733.1)	*Neosartorya fischeri* NRRL 181	86%
*gdh*1	NAD + dependent glutamate dehydrogenase, putative (XP 002144569.1)	*Penicillium marneffei* ATCC 18224	91%
	NAD + dependent glutamate dehydrogenase, putative (XP 002144568.1)	*Penicillium marneffei* ATCC 18224	95%
	NAD + dependent glutamate dehydrogenase, putative (XP 002340968.1)	*Talaromyces stipitatus* ATCC10500	94%
*gdh*2	Glutamate/Leucine/Phenylalanine/Valine/dehydrogenase, putative (XP 002341081.1)	*Talaromyces stipitatus* ATCC 10500	80%
	Glutamate/Leucine/Phenylalanine/Valine/dehydrogenase, putative (XP 002144464.1)	*Penicillium marneffei* ATCC 18224	78%
	NAD- specific glutamate dehydrogenase, putative (XP 001821419.1)	*Aspergillus oryzae* RIB40	71%

### Expression of *GS* and *Gdh* genes

**Figure 1:**
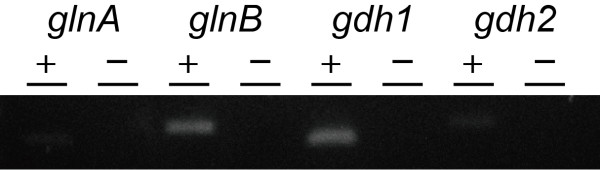
**Expression of *GS *and *Gdh *genes.** Total RNA was extracted from *P. purpurogenum* IAM15392 cultured in PP-V production medium for 72 hr. Total RNA were mixed and subjected to reverse transcription with (+) or without (−) reverse transcriptase, followed by PCR amplification. Amplification products were visualized by agarose gel (2%) electrophoresis with ethidium bromide staining.

Expression of *GS* and *Gdh* genes showed that *glnB* and *gdh1*, respectively, were expressed in PP-V production condition (Figure 1). In contrast, *glnA* and *gdh2* amplification products produced only faint bands on agarose gel, demonstrating that *glnA* and *gdh2* are expressed at very low levels under the conditions of PP-V production.

**Figure 2:**
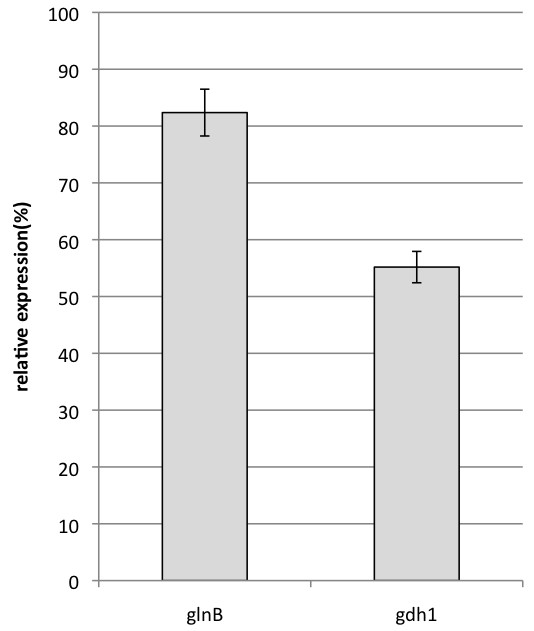
**Expression of *glnB *and *gdh1*.***P. purpurogenum* IAM15392 was cultured in PP-V production medium for 72 hr. After total RNA extraction, qRT-PCR was carried out using the LightCycler Fast Start DNA Master SYBR Green I kit on a LightCycler.

Examination of *glnB* and *gdh1* expression levels by qRT-PCR showed that *GlnB* expression was slightly higher than that of *gdh1*, but the differences could not be clearly differentiated (Figure 2).

### Effect of Gln and Glu supplementation on PP-V production

**Figure 3:**
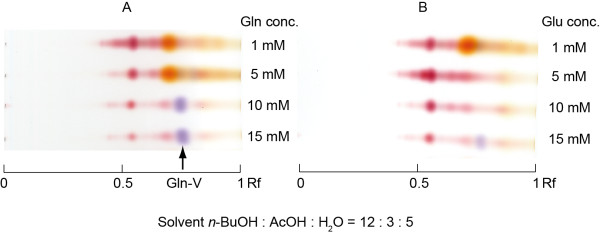
**Silica gel TLC profiles of culture filtrate pigments and the effect of Gln and Glu on pigment production.** (**A**) PP-V production medium containing 1 to 15 mM L-glutamine. (**B**) PP-V production medium containing 1 to 15 mM L-glutamate. Pigments were extracted from cultures incubated for 120 hr with EtOAc and separated on silica gel TLC using developing solvents *n*-BuOH:AcOH:H_2_O (12:3:5). The violet pigment at Rf 0.75 was identified as PP-V. Smaller compound than Gln-V (Rf = 0.75) was observed. In our other researches, Smaller compound was considered Amino acid derivatives of *Monascus* pigment homologs (date not shown).

The effects of Gln and Glu supplementation on pigment production are shown in Figure 3. The violet pigment, Rf, was observed at Rf 0.75 (Ogihara et al. 2000). When cultured with Gln, the violet pigment, Gln-V, was observed at concentration of 10 and 15 mM and increased with increasing Gln concentration. However, violet pigment was observed only for cultures supplied with 15 mM Glu. Gln supplementation stimulated Gln-V production much more effectively than did Glu (Figure 3).

### NMR analysis of violet pigment

In the purification of the major violet pigment, Gln-V, a yield of at least 0.3 g was obtained from a 2.5-L culture following the first silica gel column chromatography step. The yield of the purified preparation for NMR analysis was 3.4 mg. Gln-V is a blue-black powder. The ^1^H and ^13^C NMR spectral data for Gln-V determined here and PP-V (Ogihara et al. 2000) are shown in Table 3. The results of NMR analyses indicate that the chemical shift value of Gln-V is in accordance with that of PP-V. The coupling constant between H-10 and H-11 (13.0 Hz) indicated the presence of a *Z* configuration. The stereochemistry of the double bond at C-10 was further elucidated by differential nuclear Overhauser effects (NOE). The relationship among H-5, H-10, and H-11 also confirmed the *Z* configuration. Violet pigment Gln-V was thus identified as PP-V [(10*Z*)-12-carboxyl-monascorubramine].

**Table 3:** ^**13**^**C and **^**1**^**H NMR spectral date of Gln-V and PP-V**

	**Gln-V**		**PP-V**	
**Pos. No.**	**δ**_**C**_	**δ**_**H**_	**δ**_**C**_	**δ**_**H**_
2	171.7		171.2	
3	97.8		97.5	
3a	169.0		168.6	
4	96.1	6.45(1H, s)	95.7	6.49(1H, s)
4a	152.9		152.5	
5	123.0	7.12(1H, s)	122.5	7.16(1H, s)
6	152.0		151.4	
8	143.9	8.34(1H, s)	143.3	8.37(1H, s)
8a	118.5		117.9	
9	197.9		197.4	
9a	85.0		84.6	
9a-CH3	30.9	1.46(3H, s)	30.4	1.50(3H, s)
10	135.4	6.76(1H, d, *J* = 13.0)	134.7	6.78(1H, d, *J* = 13.2)
11	131.2	6.76(1H, d, *J* = 13.0)	130.9	6.09(1H, d, *J* = 13.2)
12	167.1		166.8	
13	193.9		193.5	
14	39.7	2.63(2H, t, *J* = 7.3)	39.2	2.65(2H, t, *J* = 7.3)
15	25.2	1.44(2H, quintet, *J* = 7.3)	24.7	1.46(2H, quintet, *J* = 7.3)
16	29.6	1.20-1.23(8H, m)	29.1	1.23-1.25(8H, m)
17	29.2	1.20-1.23(8H, m)	28.7	1.23-1.25(8H, m)
18	30.8	1.20-1.23(8H, m)	31.8	1.23-1.25(8H, m)
19	22.6	1.20-1.23(8H, m)	22.1	1.23-1.25(8H, m)
20	14.5	0.82(8H, t, *J* = 7.0)	14.0	0.84(8H, t, *J* = 7.0)

Measured in DMSO-*d*_6_.

125 MHz for ^13^C and 500 MHz for ^1^H.

### Effect of GS inhibition on pigment production

**Figure 4:**
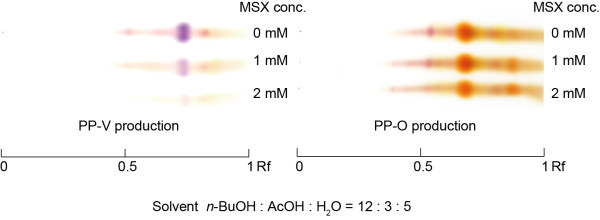
**Silica gel TLC profiles of culture filtrate pigments and the effect of GS inhibitor on pigment production.** PP-V or PP-O production medium cultures containing 0, 1 and 2 mM MSX were cultured for 72 hr. Pigments were extracted with EtOAc and resolved on silica gel TLC using developing solvent *n*-BuOH:AcOH:H_2_O (12:3:5). Violet pigment at Rf 0.75 was identified as PP-V. Orange pigment at Rf 0.65 was identified as PP-O.

The effect of GS inhibition on pigment production is shown in Figure 4. The amount of PP-V was markedly decreased with increasing GS-inhibitor, MSX, concentration. In contrast, PP-O production was little affected by MSX (Figure 4), and no inhibition of growth of *P. purpurogenum* IAM15392 or PP-O production was observed up to 2 mM MSX.

## Discussion

*P. purpurogenum* IAM15392 has been found to produce amino derivatives of *Monascus* pigment homolog, PP-V, in culture with a medium containing ammonium nitrate (Ogihara et al. 2000). Here, we investigated whether ammonium that is transported into the cell acts directly or is incorporated into amino acids prior to PP-V production.

Ammonia taken into the cell is converted to Glu and Gln through an ammonia assimilation process catalyzed in fungi by Gdh to form Glu, or by GS to form Gln (Hammond and Wood 1985 ; Casper et al. 1985 ; Dunn-Coleman et al. 1981 ; Limon-Lason et al. 1977). Therefore, we examined the genes coding for GS and Gdh in *P. purpurogenum* IAM15392, and their expression. Genes from other filamentous fungi were used to produce specific primers for amplification and showed significant homology to *glnA* and *glnB* of *GS* and *gdh1* and *gdh2* of *Gdh* in *P. purpurogenum* IAM15392 (Table 2)*.* RT-PCR demonstrated that *glnB* and *gdh1* are expressed in culture conditions conducive to PP-V production. Further, *glnB* and *gdh1* expression levels show no marked differences in expression, suggesting that both are involved in ammonia assimilation.

The violet pigment produced by *P. purpurogenum* IAM15392 was identified as PP-V, and the addition of Gln and Glu as nitrogen sources stimulated its production, though Gln had a much greater effect than Glu. Therefore, we conclude that the ammonia assimilation by GS is important in PP-V production. Gln is converted to Glu and ammonia by glutaminase. Glutaminases have been identified in bacteria, yeast, fungi, and mammals, and they play an important role in nitrogen metabolism (Durá et al. 2002 ; Heini et al. 1987 ; Imada et al. 1973). We consider that the ammonia generated by glutaminase in these cultures is used in PP-V production. Amino acid derivatives of *Monascus* pigments are known to be produced as water-soluble pigment by *Monascus* spp. when amino acids are added to the culture medium (Lin et al. 1992 ; Blanc et al. 1994 ; Hajjaj et al. 1997 ; Sato et al. 1997 ; Jung et al. 2003). In this study, *P. purpurogenum* IAM15392 produced an amino derivative pigment, PP-V, and as an EtOAc-soluble pigment when Gln or Glu are added to the culture medium. Hence, we consider that *P. purpurogenum* IAM15392 and *Monascus* spp. utilize a different nitrogen source for pigment production.

MSX is known to inhibit GS of fungi, yeasts and plants, and it is used to examine the influence of GS on secondary metabolite biosynthesis on GS in the filamentous fungus (Muñoz and Agosin 1993). Here, PP-V production was reduced, but not completely inhibited, by MSX. For the addition of Glu, a small quantity of PP-V was produced. Therefore, it is thought that Glu formed from ammonia by Gdh is also used in PP-V production. However, the decrease in PP-V production by MSX is remarkable. Hence, we demonstrate here that the glutamine synthesis of ammonium by GS is a critical reaction in the PP-V production pathway.

We conclude that ammonia is utilized as a nitrogen source in the production of PP-V after first being incorporated into amino acids. The nitrogen atom in the nitro-compound contributes to the expression of various biological activities in medical and agrochemical compounds. The next area of research will be to identify the enzymes that actually introduce nitrogen into the pyranoid ring of PP-O.

## Competing interests

The authors declare that they have no competing interests.
